# Bottled water brands are contaminated with multidrug resistant bacteria in Nairobi, Kenya

**DOI:** 10.12688/f1000research.24031.2

**Published:** 2021-03-03

**Authors:** Safia Adam Mohamed, Andrew Nyerere, Willie Kipkemboi Sang, Musa Ngayo

**Affiliations:** 1 Institute of Tropical Medicine and Infectious Diseases, Jomo Kenyatta University of Agriculture and Technology, Nairobi, Kenya; 2 Centre for Microbiology Research, Kenya Medical Research Institute, Nairobi, Kenya, Kenya

**Keywords:** Bottled water brands, Bacteriological quality, multi-drug resistant bacteria, role of water companies handling procedures

## Abstract

**Background:** The demand for drinking water has necessitated the proliferation of bottled water companies in Kenya. This study evaluated if retailed bottled water in Nairobi Kenya complies with both local and international reference criteria.

**Methods:** A total of 42 different water brands (25 approved by Kenya Revenue Authority (KRA) and 17 banned brands) were analyzed for both physicochemical and bacteriological quality. The spread plate method was used to obtain the total plate count of bacteria, while the membrane filter method was used to obtain total coliform count (TCC) and fecal coliform count (FCC). Structured interviews were used to gather company-related information.

**Results:** Overall, 16% of KRA-approved and 35.3% of banned bottled water were contaminated with heterotrophic bacteria. Of the approved water brands, 4% were positive for total coliforms, compared with 17% of the banned brands.  Similarly, 4% and 17% approved and banned water brands were positive for fecal coliforms, respectively. *Escherichia coli* (19.1%), *Pseudomonas* spp. (9.5%) and *Klebsiella* spp. (4.8%) were the most common bacterial types isolated from all water brands, most of which exhibited multidrug resistance. In multivariable analysis, water companies that cleaned pipework and bottles using chlorine-based disinfectants (OR 0.08, 95% CI 0.01 to 0.8), those that had food safety programs (OR 0.1, 95% CI 0.019 to 0.9), had standard operating procedures (SOP) for water sourcing (OR 0.1, 95% CI 0.012 to 0.9) and SOP for contamination protection (OR 0.1, 95% CI 0.02 to 0.9) remained independently associated with bottled water brands exceeding WHO TCC limits.

**Conclusions:** A number of bottled water brands were contaminated with one or more types of indicator bacteria, some of which were multidrug-resistant. Water bottling companies’ processes contribute to contamination. Rigorous regulation and monitoring will improve water quality and safety.

## Introduction

Water is essential for the human body and mental functions ^1– 3^ as well as for chronic disease prevention ^4^. Water is essential for thermoregulation, protection and cushioning of body vital organs, as well as for breathing and transporting nutrients and oxygen throughout the body ^2^. It is not surprising therefore that water constitute 50–60% of the human body ^2^. Inevitably therefore, adequate total water intake of between 2 to 2.5 liters per day is recommended ^2^.

Achieving and maintaining good health requires the availability and consumption of clean, potable (drinkable) water. This requires that water must be devoid of pathogens, dissolved toxins, and disagreeable turbidity, odor, color and taste ^5^. The current concerns about palatability and microbial and chemical contaminants in tap water ^6^, have led to the proliferation in the consumption of bottled water reaching historical high accounting for billion gallons in consumption ^6^. Bottled water offers a handy source of water for consumption both within and outside household settings. In developing countries such as Kenya, bottled water is habitually sold and consumed in hotel industries, markets places, streets, schools, and during mass gatherings such as wedding and spotting activities, workplaces, health care facilities, and emergency situations ^7^. Unfortunately, bottled water is not always as sterile as perceived. Several reports are available showing contamination bottled water with heterotrophic bacteria and coliforms counts exceeding the national and international standards ^8, 9^. Studies have isolated various bacterial contamination from bottled water such as *Vibrio cholera* and *Salmonella* spp. ^10, 11^, *Pseudomonas* spp., *Acinetobacter* spp., *Citrobacter* spp. and *C. violaceum* ^9^. As a result, several waterborne illnesses such as diarrhea account for significant morbidity and mortality among the young and the aged as well as immunocompromised populations ^12, 13^.

The bottled drinking water in Kenya should meet the following minimum requirements: be free from pathogens and chemicals; clear (i.e. low turbidity); none saline and should not have offensive taste or smell ^14^. The Kenyan Bureau of standard (KS EAS 153: 2014) reference criteria for packaged water requires the absence of total coliforms, *Escherichia coli*, *Staphylococcus aureus*, *Pseudomonas aeruginosa*, *Streptococcus faecalis*, *Shigella* and *Salmonella* in 100 ml of water ^14^. Microbiological contamination of drinking water can have an immediate and significant impact on human health and must therefore be analyzed frequently. Among the factors reported to influence the microbiological quality of bottled water include; material of bottles, color of bottles and the length of storage ^15^. This study investigated the bacteriological quality of bottled water and the association with the processes and handling practices of water bottling companies sold in Nairobi Kenya.

## Methods

### Ethical statement

This study was approved by Kenyatta National Hospital and University of Nairobi Ethical Review Committee (KNH-UoN ERC-P971/12/2016). Before recruitment to this study, all study participants provided written informed consent for study participation.

### Study design

This descriptive cross-sectional study was carried out at the Center for Microbiology Research (CMR), Kenya Medical Research Institute (KEMRI) (International Organization for Standardization [ISO] 9001:2008– certified) between February 2019 and January 2020.

***Sample size.*** There are about 40 registered water bottling companies in Nairobi ( http://www.businesslist.co.ke/category/bottled-water/city:nairobi). Further, the Kenya revenue authority (KRA) has listed about 369 banned water bottling companies at https://www.slideshare.net/starwebmaster/list-of-368-water-brands-banned-by-kebs. To select the bottled water samples in this study, we used the 26% failure rate of the bottled water brands in Nigeria ^16^ to meet the United States Environmental Protection Agency (USEPA) and World Health Organization (WHO) requirement for drinking water standard of 100 total coliforms/ml water. Applying the formula for estimating the population proportion with specified relative precision described by Lemeshow *et al*. ^17^, setting the α at 0.05, a total of 288 bottled water samples were collected to achieve 0.90 power. This number of bottled water samples was divided equally among the 40 brands sold in Nairobi. Therefore, a total of seven bottles per brand were sampled.

***Data collection.*** At the time of the study, due to availability, 25 water brands approved by KRA were purchased from major retail outlets in Nairobi. The other 17 water brands non-approved by KRA were purchased by the roadside or from small retail shops in the streets of Nairobi. All the brands (25 KRA-approved and 17 banned bottled water brands) were sought without preferential treatment of any brands or retail outlets. Seven bottles of each bottled water brand from the same batch were purchased at different retail outlets and shipped in cool box to the laboratory for microbiological analysis within 6 hours of purchasing.

***Structured interviews.*** To investigate the role of manufacturing handling and packaging process on the microbiological quality of water, randomly, this study visited the premises of all the available 25 registered and 17 banned water bottle and packaging companies located in Nairobi. Those consenting (see *Extended data* for the consent form 18) underwent a structured 20–30-minute face-to-face discussion within the premises at secluded and secured offices to gather information including the following information: type of abstraction, pipe work materials, bottling process, staff training, policies and procedures and microbiological quality of bottled water in Nairobi adopted from the WHO/UNICEF Joint Monitoring Programme) (see Extended data for a blank copy of the survey ^18^).

### Microbial and physicochemical quality of water samples

The water temperature and pH were measured immediately after purchase using the HACH Sensionþ MM150 Portable Multi-Parameter Meter (Hach Company, Loveland, CO), according to the manufacturer’s instructions.

Each of the seven water samples per brand were analyzed separately. Bacterial contamination in these water samples were achieved using total plate count by the spread plate method and total coliform count and fecal coliform count by membrane filter method as described by WHO, ^19^. Briefly, 100 mL of water samples were filtered through a 0.22-µm-pore-size membrane filter (Millipore Corp., Bedford, MA), and filters placed on membrane Fecal Coliform (m-FC) agar plates were incubated at 37 and 44°C for 18 to 24 h to determine total coliform (TC) and fecal coliform (FC) counts, respectively.

### Bacterial identification

The bacteria isolates were subsequently cultured onto bile esculin agar, eosine methylene blue agar, m-endo agar les, and plate count agar. These were then identified using colony morphology, Gram’s staining, biochemical tests and further characterized using the VITEK 2 system, version 0.8.01 (bioMerieux, Inc., Hazelwood, MO).

### Antimicrobial susceptibility testing

Each of the bacterial isolates were tested for susceptibility to antimicrobials by a controlled disk diffusion technique of Kirby-Bauer incubated at 35°C for 18 hours. The isolates were tested for susceptibility to the following 11 antibiotics (OXOID, England): amoxicillin (10 μg), tetracycline (30 μg), trimethoprim/sulfamethoxazole (30 μg), chloramphenicol (30 μg), gentamicin (10 μg), ciprofloxacin (5 μg), doxycycline (30 μg), erythromycin (30 μg), ofloxacin (30 μg), ceftriaxone (30 μg) and kanamycin (30 μg). These tests were done according to guidelines set by the Clinical Laboratory Standards Institute ^20^. *E. coli* ATCC 25922 (with known minimum inhibitory concentrations) was used as a reference strain in the disk diffusion susceptibility tests.

### Data analysis

Frequency (%), mean and standard deviation, were used to describe the water physiochemical properties and bacterial colony count. Chi-square or Fisher’s exact test were used to test for variation between variables. The association between the presence of TCC >100 CFU/ml contaminating bottled water and companies water handling and processing characteristics were calculated using Poisson regression. Manual backward elimination method was used to reach the most parsimonious model in multivariate analysis. This included factors that were associated with contamination with TCC >100 CFU/ml at the significance level of P≤0.05. All statistical analyses were performed using STATA v13 (StataCorp LP, College Station, TX, USA).

## Results

### Different characteristics of water samples

The buying price (mean ± SD) of KRA-approved brands was slightly higher than the banned bottled water: 37.8 ± 16.95 Kenyan shillings (Kshs) versus 29.6 ± 13.32 Kshs (p = 0.0208). The temperatures (mean ± SD) for KRA approved and banned bottled water were not significantly different 16.09 ± 0.85°C versus 16.1 ± 1.21°C, respectively (p = 0.869). On the contrary, the pH (mean ± SD) of KRA approved and banned bottled water were statistically different, at 6.8 ± 0.23 versus 7.1± 0.36, respectively (p = 0.0002). The (mean ± SD per ml) total plate count, total coliform count and fecal coliform count KRA approved bottled water were found to be lower than those from KRA banned bottled water, with values of 18.5 ± 32.89 versus 56.9 ± 122.06 (p = 0.0373), 6.9 ± 14.42 versus 33.5 ± 64.37 (p = 0.0058), and 1.02 ± 3.01 versus 14.5 ± 29.51 (p = 0.0019), respectively ( Table 1). Characteristics of each sample are available as *Underlying data* ^18^.

**Table 1.** Physiochemical properties and bacterial counts of bottled water samples approved and banned by Kenya revenue Authority.

Parameter	Kenya revenue bottled water brand approval status				*P*-value
	Approved (n = 25 brands)		Banned (n = 17 brands)		
	Range	mean ± SD	Range	mean ± SD	
**Cost**					
Buying price (Kshs)	20-65	37.8 ± 16.95	20-60	29.6 ± 13.32	0.0208
**Physiochemical properties**					
Water temp (°C)	15.1-17.8	16.09 ± 0.85	14.2-18.9	16.1 ± 1.21	0.869
pH	6.5-7.3	6.8± 0.23	6.5-8.0	7.1 ± 0.46	0.0002
**Bacterial count (CFU/mL)**					
Total plate counts	0-121	18.5 ± 32.89	0-621	56.9 ± 122.06	0.0373
Total coliforms count	0-57	6.9 ± 14.42	0-255	33.5 ± 64.37	0.0058
Fecal coliforms count	0-15	1.02 ± 3.01	0-100	14.5 ± 29.51	0.0019

Kshs, Kenyan Shillings

### Bottled water brands exceeding permitted limits on the basis of different criteria

Based on the WHO recommended criteria for drinking water, there were no KRA-approved bottled water brands exceeding the recommended pH limit of 6.5 to 7.5 while 35.3% banned bottled water exceeded the limit. With regards to total plate counts, there were 4 (16%) KRA-approved and 6 (35.3%) KRA-banned bottled water exceeding WHO criteria for drinking water. Similarly, on the basis of TCC and FCC there were 1 (4%) KRA approved and 3 (17.6%) KRA-banned bottled water brands exceeding WHO criteria for drinking water ( Table 2).

**Table 2.** Proportion of bottled water brands exceeding permitted pH, total plate count, total coliform count and fecal coliform count limits.

Parameter	WHO standards per 100 mL	Kenya Revenue Bottled Water Brand Approval status	
		Approved (n = 25 brands)	Banned (n = 17 brands)
		n (%)	n (%)
pH	6.5 - 7.5	0	6 (35.3)
Total plate counts	< 1 CFU/100mL	4 (16)	6 (35.3)
Total coliforms count	< 1 CFU/100mL	1 (4)	3 (17.6)
Fecal coliforms count	< 1 CFU/100mL	1 (4)	3 (17.6)

### Types of bacteria found contaminating different bottled water brand

*E. coli* was the most common bacteria type found contaminating four (16%) different KRA-approved bottled water brands and four (23.5%) of the banned brands. Other bacteria isolated from the KRA-approved bottled water brands included *Pseudomonas* spp. (n=4, 16%), *Enterobacter* spp. (n=1, 4%), *Klebsiella* spp. (n=1, 4%) and *Proteus* spp. (n=1 (4 %). With regards to KRA banned bottled water samples apart from the *E. coli*, other isolated bacteria were *Enterobacter* spp. (n=1, 5.9%), *Klebsiella* spp. (n=1, 5.9%) and *Aeromonas* spp. (n=1, 5.9%).

### Antimicrobial resistance patterns of bacteria contaminating bottled drinking water

Susceptibility testing showed that all the bacterial isolates were resistant to at least one type of antibiotics. All the isolates were susceptible to ceftriaxone and ofloxacin. Most of the bacterial isolates 19 out of 28 (67.9%) were resistant to amoxicillin. The bacteria isolates were also resistance to erythromycin (14/28; 50%), trimethoprim-sulfamethoxazole (8/28; 28.6%), doxycycline (7/28; 25%), tetracycline (5/28; 17.9%), gentamycin (4/28; 14.3%), chloramphenicol (4/28; 14.3%), kanamycin (3/28; 10.7%) and ciprofloxacin (3/28; 10.7%).

Most bacteria from KRA-banned bottled waters were resistant to gentamycin and erythromycin. The bacteria from the KRA approved brands were mostly resistant to trimethoprim-sulfamethoxazole, Amoxicillin, tetracycline and doxycycline. Most of multidrug resistance (resistant to more than three drugs) *E. coli* and *Klebsiella* spp. were from KRA-banned bottled water, while multidrug resistance *Pseudomonas* spp. from KRA-approved brands ( Table 3).

**Table 3.** The antibiotic resistance profiles of all bacterial isolates from bottled water brands.

		Antimicrobial types											
Pathogen	KRA approval	GEN	STX	C	AML	K	TET	OFX	CIP	E	DXT	CHLO	N (%) Resistant
*E. coli*	Banned	R	S	R	R	S	S	S	S	R	S	S	4
*E. coli*	Banned	S	S	S	R	S	S	S	S	R	R	S	3
*E. coli*	Banned	S	R	S	R	S	S	S	S	R	S	S	3
*E. coli*	Banned	S	R	S	S	R	S	S	S	S	S	S	2
*E. coli*	Banned	S	S	S	R	S	S	S	S	R	R	S	3
*E. coli*	Banned	S	S	S	R	S	S	S	S	R	S	S	2
*Enterobacter* spp.	Banned	S	R	S	S	S	S	S	S	R	S	S	2
*Klebsiella* *ozaenae*	Banned	S	S	S	S	S	S	S	S	R	S	S	1
*Klebsiella* *ozaenae*	Banned	R	S	S	R	R	R	S	R	S	S	S	4
*Aeromonas* spp.	Banned	R	S	S	R	S	S	S	S	S	S	S	2
*Aeromonas* spp.	Banned	S	S	R	S	S	S	S	S	R	S	S	2
	**Subtotal**	**3**	**3**	**2**	**7**	**2**	**1**	**0**	**1**	**8**	**2**	**0**	
*E. coli*	Approved	S	R	S	R	S	S	S	S	S	S	S	2
*E. coli*	Approved	S	S	S	R	S	S	S	S	R	S	S	2
*E. coli*	Approved	S	S	S	S	S	R	S	S	R	R	S	3
*E. coli*	Approved	S	S	S	R	S	S	S	S	S	S	S	1
*E. coli*	Approved	S	S	S	R	R	S	S	S	R	S	S	3
*E. coli*	Approved	S	R	S	S	S	S	S	S	S	R	S	2
*Enterobacter* spp.	Approved	S	S	S	R	S	S	S	S	S	S	S	1
*Proteus* spp.	Approved	S	S	R	S	S	S	S	S	S	S	S	1
*Proteus* spp.	Approved	S	R	S	R	S	S	S	S	S	S	S	2
*Klebsiella* *pneumonia*	Approved	S	S	S	R	S	R	S	S	S	S	S	2
*Klebsiella* *pneumonia*	Approved	S	R	S	S	S	R	S	S	S	R	S	3
*Pesudomonas* *putida*	Approved	S	S	R	R	S	S	S	S	S	S	S	2
*Pseudomonas* *putida*	Approved	R	S	S	R	S	R	S	R	S	S	S	4
*Pseudomonas* *putida*	Approved	S	R	S	R	S	S	S	S	S	R	S	3
*pseudomonas* spp.	Approved	S	S	S	S	S	S	S	S	R	S	S	1
*pseudomonas* spp.	Approved	S	S	S	R	S	S	S	S	R	S	S	2
*pseudomonas* spp.	Approved	S	S	S	R	S	S	S	R	R	R	S	4
	**Subtotal**	1	5	2	12	1	4	0	2	6	5	0	
**N (%)**	**Total** **Resistant**	**4** **(14.3)**	**8** **(28.6)**	**4** **(14.3)**	**19** **(67.9)**	**3** **(10.7)**	**5** **(17.9)**	**0**	**3** **(10.7)**	**14** **(50)**	**7** **(25)**	**0**	**N (%)**

Each row represents one different bacterial isolate. GEN, gentamicin; SXT, trimethoprim-sulfamethoxazole; C, ceftriaxone; AML, amoxicillin; K, kanamycin; TE, tetracycline; OFX, ofloxacin; CIP, ciprofloxacin; E, erythromycin; DXT, doxycycline; CHLO, chloramphenicol

### Company-related factors associated with acceptability of water samples

In multivariable analysis, bottled water brands that used chlorine-based disinfectants for cleaning pipework/tankers and bottling equipment were less likely to exceed WHO TCC limits compared to those that did not use any detergent for cleaning (OR 0.08, 95% CI 0.007 to 0.8). Companies that had food safety programs (OR 0.1, 95% CI 0.019 to 0.9), procedures for water sourcing (OR 0.1, 95% CI 0.012 to 0.9) and procedures for contamination protection (OR 0.1, 95% CI 0.02 to 0.9) ( Table 4). Self-reported company details are available as *Underlying data* ^18^.

**Table 4.** Company related factors influencing acceptability of water samples.

			Bottled water exceeding WHO TCC limits		
Variables	Unit	Total	n (%)	uOR (95% CI)	aOR (95% CI)
KRA approval	Approved	24	1 (4.2)	0.3(0.03 - 2.4)	0.2(0.02 - 2.9)
	Banned	18	3(16.7)	Referent	Referent
Water source	Borehole	23	1(4.3)	0.3(0.03 - 2.9)	0.2(0.02 - 3.2)
	Pipped	19	3 (15.8)	Referent	Referent
Backflow device installed	Yes	36	1 (2.8)	**0.6 (0.07 - 0.5)**	0.5(0.03 - 9.4)
	No	6	3 (50)	Referent	Referent
Water treatment	Filtration and UV	21	2 (9.5)	1.3(0.2 - 14.7)	2.9(0.2 - 39.3)
	Filtration, UV and Chlorination	15	1 (7.1)	Referent	Referent
	Filtration-reverse osmosis	6	1 (14.3)	2(0.1 - 31)	1.5(0.09 - 24.9)
Types of bottles used	Plastics	22	2 (9.1)	1.1(0.2 - 7.8)	4.9(0.4 - 66.8)
	Polycarbonate	20	2(10)	Referent	Referent
Packaging volumes	5L, 10L and 20L	7	1 (14.3)	1.6(0.2 - 18.1)	0.8(0.07 - 8.7)
	300ml, 500ml, 1L, 5L and 20L	12	1 (8.3)	0.9(0.08 - 10.6)	0.03(0.005 - 5.8)
	300ml, 500ml, 1L, 1.5l, 5L, 10L and 20L	23	2 (8.7)	Referent	Referent
Cleaning type for pipework/tankers/ bottling equipment	Active ozone	4	1 (25)	0.6(0.05 - 6.8)	0.6(0.06 - 6.9)
	Chlorine based disinfectants	32	1(3.1)	**0.07(0.01 - 0.8)**	**0.08(0.007 - 0.8)**
	None	6	3(33.3)	Referent	Referent
Batch tracing system	Yes	22	1(4.5)	0.3(0.03 - 2.9)	NS
	No	20	2(10)	Referent	
Food Safety program	Yes	36	1 (2.8)	**0.06(0.006 - 0.5)**	**0.1(0.019 - 0.9)**
	No	6	3 (50)	Referent	Referent
Tests routinely undertaken	TCC, *E. coli*, *Pseudomonas* *aeruginosa* and Yeasts & mold	12	0	NS	
	TCC, *E. coli* and Enterococci	4	1 (25)	1.3(0.1 - 15.2)	NS
	TCC and *E. coli*	15	1 (6.7)	0.4(0.03 - 4.1)	
Number of staffs	1 to 10	13	1 (7.7)	0.7(0.08 - 7.1)	0.1(0.005 - 2.6)
	>11	29	3 (10.3)	Referent	Referent
Staff qualifications	Secondary	32	3 (9.4)	0.9(0.09 - 9.1)	0.4(0.02 - 7.9)
	Tertiary	10	1 (10)	Referent	Referent
Availability of procedures for water sourcing	Yes	30	1 (3.3)	**0.1(0.02 - 0.9)**	**0.1(0.012 - 0.9)**
	No	12	3 (25)	Referent	Referent
Availability of procedures for water bottling	Yes	28	1 (3.6)	0.1(0.01 - 1.2)	NS
	No	14	3 (21.4)	Referent	
Availability of procedures for water delivery and dispatch	Yes	34	1 (2.9)	**0.07(0.008 - 0.8)**	0.1(0.009 - 1.9)
	No	8	3 (37.5)	Referent	Referent
Availability of procedures for contamination protection	Yes	33	1 (3)	**0.09(0.009 - 0.9)**	**0.1(0.02 - 0.9)**
	No	9	3 (33.3)	Referent	Referent
Availability of procedures on staff health	Yes	37	2(5.4)	**0.1(0.02 - 0.9)**	0.4(0.03 - 3.9)
	No	5	2(40)	Referent	Referent
Common problems faced by the company	Chlorination and fluoride water levels	10	1 (10)	0.7(0.05 - 7.2)	
	Counterfeits	12	0	NS	NS
	Sewage contamination	7	1 (14.3)	0.9(0.08 - 10.2)	
	Stiff competition	13	2 (15.4)	Referent	

OR, odds ratio; CI, confidence interval; NS – not significant/done; uOR, Unadjusted OR; aOR, adjusted OR.

## Discussion

Evaluation of bacteriological quality of bottled drinking water is important and urgent in Kenya given the current upsurge of different brands of bottled water, most of which are not regulated. This study was unique and among the first in Kenya to evaluate the role of the practices used by water bottling companies in relation to the bacterial quality of water in line with the WHO acceptability criteria. This was compared between those bottled waters approved and banned brands by Kenya Revenue Authority (KRA). The bacteriological quality of bottled water from approved brands was found to be better than those of banned brands. The total coliforms and fecal coliform present in 100 ml of water were detected cumulatively in 9.5% of all brands, and in 4% of KRA-approved and 17.6% of banned bottled water brands. The proportion of bottle water brands with unacceptable in line with WHO limits were lower than the 50% reported in Bangladesh ^21^, 37.5% in India ^22^, 26% reported in Nigeria ^16^ and 25% in Nepal ^9^. On the contrary, the proportion of unacceptable bottled water brands in our study was higher than the 4.6% reported in Tanzania ^23^ the 9% in Sri Lanka ^24^ and 0% reported in Saudi Arabia ^25^. Although KRA approval is based on tax payment rather than on scientific basis, the high number of KRA-banned bottled water brands points to the possibilities of ineffectiveness of the disinfection processes used in these brands. In a process likely to be mainly for financial benefit by the bottled water manufacturers, studies have cited the improper practice of filling the bottle directly from tap water and sealing it without any prior treatment as among the reasons responsible for higher brands of bottled water beyond the acceptable limits of bacteriological quality ^9^. Longer storage periods, especially of already-contaminated bottled water, have been shown to worsen the bacteriological quality. As in many developing countries, the laxity by the government body responsible for monitoring the quality of bottled water has been shown to account for higher levels of bottled water brands with unacceptable microbiological limits ^9^.

With regards to total plate count or heterotrophic bacteria, in this study, a total of 23.8% of the bottled water brands (40% KRA-approved and 60% banned brands) were contaminated. In other settings, higher percentages of between 20% to 100% of heterotrophic bacteria contamination of bottled drinking water have been reported ^9, 26, 27^. Studies have associated long storage duration with high levels of bacterial concentration mainly due to larger surface area for growth, higher temperature, and the nutrients arising in the container ^28^. This quantity of heterotrophic bacteria is shown to correlate with water pH. There were 35.5% of banned bottled water brands with a pH below the pH 6.5 minimum level recommended by WHO, which could account for the higher numbers of heterotrophic bacteria per milliliter detected in these brands. Similar results were also reported by Pant *et al*. ^9^.

The presence of total coliforms and fecal coliforms in 4% KRA-approved and 17.6% KRA-banned bottled water brands exceeding WHO criteria is similar to that observed by other studies ^23, 29^. This is worrying and a pointer to either poor water processing, introducing flakes of human skin or indigenously acquired by filling the bottles directly from the natural sources or taps ^23^.

*E. coli* (in 19.1%) was the most common bacteria found contaminating the bottled water brands. Others included *Pseudomonas* spp. (9.5%), *Enterobacter* spp. (4.8%), *Klebsiella* spp. (4.8%) and *Proteus* spp. (2.3%) and *Aeromonas* spp. (2.3%). In Nepal, Pant *et al*. ^9^ isolated more of *Pseudomonas* spp. (87.5 %) and *Acinetobacter* spp. (87.5 %). In Iran Momtaz *et al.*, ^10^ isolated *E. coli*, while in Brazil, Vasconcellos *et al*. ^30^ isolated *Salmonella* spp., and *V. cholerae* from bottled water.

Although all bacteria in our investigation were susceptible to ceftriaxone and ofloxacin, resistance to erythromycin, trimethoprim-sulfamethoxazole, doxycycline, tetracycline, gentamycin, chloramphenicol, kanamycin and ciprofloxacin were noted. Multidrug resistance (resistant to more than three drugs) in *E. coli*, Klebsiella spp. and *Pseudomonas* spp. from all bottle brands were also detected. The presence of different species of bacteria, including multidrug resistant strains, in supposedly bacteria-free bottled water is of important public health problem. Pathogenicity notwithstanding, their presence in bottled waters heavily consumed by those including the elderly, children and the immunocompromised, the hazards of contamination, and health risks to consumers should not be taken for granted ^10, 30^.

This study incorporated a unique feature by investigating the manufacturing practices potentially associated with contamination of bottled water. In multivariable analysis, companies that used chlorine-based disinfectants for cleaning pipework/tankers and bottling equipment were less likely to have water brands exceeding WHO TCC limits compared to that that did not use any detergent for cleaning. Zamberlan *et al*. ^31^ showed the importance of disinfection processes used by the water bottling companies as playing a key determinant of bacterial concentration in bottled water. In Nepal, Pant *et al*. ^9^ showed that failure to disinfect water represents an important avenue for bacterial entry and colonization of water processing systems. Our study further showed that companies that had food safety programs, procedures for water sourcing and procedures for contamination protection were less likely produce bottled waters with unacceptable microbiological limits. As expected, if companies are set up in line with guidelines set by regulatory authorities, then the end product will be devoid or have a reduced microbial contamination. In this study, although more of companies producing approved brands had recommended water collection and transportation systems, water treatment procedures (filtration, UV and chlorination and reverse osmosis), packaged water using polycarbonate containers, used machine during bottling process, had batch tracing system, routinely tested their products according to the WHO guidelines and having recommended handling standard procedures than banned ones, these were not factors associated with the contamination of bottled water.

Our study had some limitations. First, due to limited resources available, the study could not process large enough numbers of the samples to include all brands sold in the country. Second, owing to the limited laboratory methods used, we were not able to identify all the potential pathogens that contaminated the water, including other pathogenic bacteria, viruses, fungi, and parasites. Third, the cross-sectional nature of our study only allowed us to describe associations between water company processes and procedures and bacterial quality and not a causal conclusion. Such outcomes can be confirmed in a longitudinal study. These limitations notwithstanding, one of the key outcomes of this investigation is the capacity to show that the perceived safe bottled water brands, including the top-selling and most expensive brands in Kenya, could be contaminated with bacteria beyond the WHO recommended limits. Additionally, some of these bacteria associated with significant disease outbreaks were multidrug-resistant. The study also showed that water bottling companies’ operations and processes are key avenue for bacterial water contamination. The Kenya Bureau of Standards is the Kenyan regulatory and monitoring authority for all water and packaged foods. Our results may suggest, however, that concerted efforts must be made to improve the ability of national governments to properly regulate and monitor these products which has been shown to improve product quality and safety ^32, 33^.

## Data availability

### Underlying data

Figshare: Bottled water brands are contaminated with multidrug resistant bacteria which are associated with companies handling procedures in Nairobi Kenya. https://doi.org/10.6084/m9.figshare.13046534.v2 ^18^

This project contains the following underlying data:

- Safia Company Response F1000R.xlsx. (Company responses to each question of the survey.)
- Safia Water property F1000 Data 1.xlsx. (Properties of each bottled water sample analyzed in this study.)

### Extended data

Figshare: Bottled water brands are contaminated with multidrug resistant bacteria which are associated with companies handling procedures in Nairobi Kenya. https://doi.org/10.6084/m9.figshare.13046534.v2 ^18^

This project contains the following extended data:

- Safia F1000_Consent.docx. (Informed consent form.)
- Safia F1000 Interview guide.docx. (Survey used in the present study.)

Data are available under the terms of the Creative Commons Attribution 4.0 International license (CC-BY 4.0).

## References

[1] Popkin BM D’Anci KE Rosenberg IH: Water, hydration, and health. *Nutr Rev.* 2010;68(8):439–458. 10.1111/j.1753-4887.2010.00304.x 20646222 PMC2908954

[2] EFSA Panel on Dietetic Products Nutrition and Allergies (NDA): Scientific Opinion on the substantiation of health claims related to water and maintenance of normal physical and cognitive functions (ID 1102, 1209, 1294, 1331), maintenance of normal thermoregulation (ID 1208) and “basic requirement of all living things. *EFSA J.* 2011;9(4):2075. 10.2903/j.efsa.2011.2075 PMC13129695 42078698

[3] Pross N Demazières A Girard N: Influence of progressive fluid restriction on mood and physiological markers of dehydration in women. *Br J Nutr.* 2013;109(2):313–321. 10.1017/S0007114512001080 22716932 PMC3553795

[4] Muckelbauer R Sarganas G Grüneis A: Association between water consumption and body weight outcomes: A systematic review. *Am J Clin Nutr.* 2013;98(2):282–299. 10.3945/ajcn.112.055061 23803882

[5] Gazan R Sondey J Maillot M: Drinking Water Intake Is Associated with Higher Diet Quality among French Adults. *Nutrients.* 2016;8(11):689. 10.3390/nu8110689 27809236 PMC5133077

[6] Kohnen W Teske-Keiser S Meyer HG: Microbiological quality of carbonated drinking water produced with in-home carbonation system. *Int J Hyg Environ Health.* 2005;208(5):415–423. 10.1016/j.ijheh.2005.04.008 16217926

[7] Williams AR Bain RE Fisher MB: A Systematic Review and Meta-Analysis of Fecal Contamination and Inadequate Treatment of Packaged Water. *PLoS One.* 2015;10(10):e0140899. 10.1371/journal.pone.0140899 26505745 PMC4624706

[8] Semerjian LA: Quality assessment of various bottled waters marketed in Lebanon. *Environ Monit Asses.* 2011;172(1–4):274–85. 10.1007/s10661-010-1333-7 20148363

[9] Pant ND Poudyal N Bhattacharya SK: Bacteriological quality of bottled drinking water versus municipal tap water in Dharan municipality, Nepal. *J Health Popul Nutr.* 2016;35(1):17. 10.1186/s41043-016-0054-0 27267213 PMC5025974

[10] Momtaz H Dehkordi FS Rahimi E: Detection of *Escherichia coli*, Salmonella species, and *Vibrio cholerae* in tap water and bottled drinking water in Isfahan, Iran. *BMC Public Health.* 2013;13:556. 10.1186/1471-2458-13-556 23742181 PMC3703282

[11] Beuret C Kohler D Baumgartner A: Norwalk-LikeVirus Sequences in Minelral Waters: One-Year Monitoring of Three Brands. *Appl Environ Microbiol.* 2002;68(4):1925–1931. 10.1128/aem.68.4.1925-1931.2002 11916714 PMC123876

[12] WHO, UNICEF: Progress on drinking water and sanitation: 2015 update and MDG assessment.2015. Reference Source

[13] WHO: Mortality and burden of disease from water and sanitation. 2018; Accessed January, 2020. Reference Source

[14] Kenya Bureau of Standards: Kenya standard Potable water — Specification.2015; accessed on 15th November, 2019. Reference Source

[15] Akond MA Alam S Shil A: Bacteriological quality of bottled water available commercially in Bangladesh. *Journal of Environmental Sciences (Dhaka).* 2006;4:47–52. Reference Source

[16] Igbeneghu OA Lamikanra A: The bacteriological quality of different brands of bottled water available to consumers in Ile-Ife, south-western Nigeria. *BMC Res Notes.* 2014;7:859. 10.1186/1756-0500-7-859 25432739 PMC4307168

[17] Lemeshow S Hosmer DW Jr Klar J: Sample size requirements for studies estimating odds ratios or relative risks. *Stat Med.* 1988;7(7):759–764. 10.1002/sim.4780070705 3406603

[18] Mohamed SA Nyerere AS Willie K: Bottled water brands are contaminated with multidrug resistant bacteria which are associated with companies handling procedures in Nairobi Kenya. *figshare.*Dataset2020. 10.6084/m9.figshare.13046534.v2

[19] World Health Organization: Guidelines for drinking-water quality. 4th ed. Geneva: WHO;2011; accessed January 2020. Reference Source

[20] Clinical and Laboratory Standard Institute (CLSI): Performance Standards for Antimicrobial Disk Susceptibility Tests. 13th ed. Wayne, Pennsylvania 19087 USA, 2018. Reference Source

[21] Islam S Begum HA Nili NY: Bacteriological safety assessment of municipal tap water and quality of bottle water in Dhaka city: health hazard analysis. *Bangladesh J Med Microbiol.* 2010;4(1):9–13. 10.3329/bjmm.v4i1.8462

[22] Joseph N Bhat S Mahapatra S: Bacteriological Assessment of Bottled Drinking Water Available at Major Transit Places in Mangalore City of South India. *J Environ Public Health.* 2018;2018:7472097. 10.1155/2018/7472097 30498514 PMC6222228

[23] Kassenga GR: The health-related microbiological quality of bottled drinking water sold in Dares Salaam, Tanzania. *J Water Health.* 2007;5(1):179–85. 10.2166/wh.2006.052 17402289

[24] Sasikaran S Sritharan K Balakumar S: Physical, chemical and microbial analysis of bottled drinking water. *Ceylon Med J.* 2012;57(3):111–116. 10.4038/cmj.v57i3.4149 23086026

[25] Shahaby AF Alharthi AA El Tarras AL: Bacteriological Evaluation of Tap Water and Bottled Mineral Water in Taif, Western Saudi Arabia. *Int J Curr Microbiol App Sci.* 2015;4(12):600–615. Reference Source

[26] Khaniki GRJ Zarei A Kamkar A: Bacteriological evaluation of bottled water from domestic brands in Tehran market, Iran. *World Appl Sci J.* 2010;8(3):274–8. Reference Source

[27] Majumder AK Islam KN Nite RN: Evaluation of microbiological quality of commercially available bottled water in the city of Dhaka, Bangladesh. *Stamford J Microbiol.* 2011;1(1):24–30. 10.3329/sjm.v1i1.9099

[28] Warburton DW: The microbiological safety of bottled waters. In: Farber JM, Ewen ED T, editors. *Safe handling of foods.*New York: Marcel Dekker; 2000.

[29] Yasin N Shah N Khan J: Bacteriological status of drinking water in the peri-urban areas of Rawalpindi and Islamabad-Pakistan. *Afr J Microbiol Res.* 2012;6(1):169–75. Reference Source

[30] Vasconcellos L Medeiros VM Rosas CO: Occurrence of total coliforms, *Escherichia coli* and *Cronobacter* species in commercially available 20 l bottled drinking water sold in Rio de Janeiro State, Brazil. *Lett Appl Microbiol.* 2019;69(6):431–437. 10.1111/lam.13235 31622508

[31] Zamberlan da Silva ME Santana RG Guilhermetti M: Comparison of the bacteriological quality of tap water and bottled mineral water. *Int J Hyg Environ Health.* 2008;211(5–6):504–9. 10.1016/j.ijheh.2007.09.004 18206422

[32] Dada AC: Packaged water: optimizing local processes for sustainable water delivery in developing nations. *Global Health.* 2011;7:24. 10.1186/1744-8603-7-24 21801391 PMC3161851

[33] Stoler J Weeks JR Fink G: Sachet drinking water in Ghana’s Accra-Tema metropolitan area: Past, present, and future. *J Water Sanit Hyg Dev.* 2012;2(4):223–40. 10.2166/washdev.2012.104 24294481 PMC3842094

