# Supplementary material for: Bottled water brands are contaminated with multidrug resistant bacteria in Nairobi, Kenya
Source: F1000Res. 2021 Mar 3;9:1337. Originally published 2020 Nov 16. [Version 2] doi: 10.12688/f1000research.24031.2 (PMC7970434; doi:10.12688/f1000research.24031.2)
Supplement: Peer review history

# Peer review: Reviewer response for version 2

**DOI:** 10.5256/f1000research.54975.r80749

**Published:** 2021-03-17

**Authors:** Lucy A. Semerjian

**Affiliations:** 1 Department of Environmental Health Sciences, College of Health Sciences, University of Sharjah, Sharjah, United Arab Emirates

I have checked the revised manuscript and revisited the authors responses to comments.

 I approve the manuscript for indexing.

Is the work clearly and accurately presented and does it cite the current literature?

Yes

If applicable, is the statistical analysis and its interpretation appropriate?

Partly

Are all the source data underlying the results available to ensure full reproducibility?

Yes

Is the study design appropriate and is the work technically sound?

Partly

Are the conclusions drawn adequately supported by the results?

Partly

Are sufficient details of methods and analysis provided to allow replication by others?

Yes

Reviewer Expertise:

NA

I confirm that I have read this submission and believe that I have an appropriate level of expertise to confirm that it is of an acceptable scientific standard.

# Peer review: Reviewer response for version 2

**DOI:** 10.5256/f1000research.54975.r80748

**Published:** 2021-03-04

**Authors:** Ahmad Zarei

**Affiliations:** 1 Department of Environmental Health Engineering, School of Public Health, Social Development and Health Promotion Research Center, Gonabad University of Medical Sciences, Gonabad, Iran

All my comments are addressed in the paper and therefore, I propose its indexing.

Is the work clearly and accurately presented and does it cite the current literature?

Yes

If applicable, is the statistical analysis and its interpretation appropriate?

Yes

Are all the source data underlying the results available to ensure full reproducibility?

Yes

Is the study design appropriate and is the work technically sound?

Yes

Are the conclusions drawn adequately supported by the results?

Yes

Are sufficient details of methods and analysis provided to allow replication by others?

Yes

Reviewer Expertise:

NA

I confirm that I have read this submission and believe that I have an appropriate level of expertise to confirm that it is of an acceptable scientific standard.

# Peer review: Reviewer response for version 1

**DOI:** 10.5256/f1000research.26509.r74869

**Published:** 2021-02-12

**Authors:** Lucy A. Semerjian

**Affiliations:** 1 Department of Environmental Health Sciences, College of Health Sciences, University of Sharjah, Sharjah, United Arab Emirates

The research study investigates the microbial quality of selected bottled waters marketed in Kenya as well displays survey results from water bottling companies. The title needs revising as it is as a conclusion in its current language. The study is a basic investigation of microbial contamination in bottled water, drug resistance patterns of encountered microbes, and data collection on practices and processes of water bottling companies. In the Methods section, the term "patients" is invalid and needs revising as the study is not related to patients. The Sections  "Results" and "Discussion" can be combined and restructured to avoid repetition of ideas and findings. Statistical analysis and conclusions could be more detailed and justified to give the research more scientific value.

Is the work clearly and accurately presented and does it cite the current literature?

Yes

If applicable, is the statistical analysis and its interpretation appropriate?

Partly

Are all the source data underlying the results available to ensure full reproducibility?

Yes

Is the study design appropriate and is the work technically sound?

Partly

Are the conclusions drawn adequately supported by the results?

Partly

Are sufficient details of methods and analysis provided to allow replication by others?

Yes

Reviewer Expertise:

Water quality, water and wastewater treatment and reuse, emerging contaminants, PPCPs, environmental pollution control

I confirm that I have read this submission and believe that I have an appropriate level of expertise to confirm that it is of an acceptable scientific standard, however I have significant reservations, as outlined above.

# Response

**Published:** 2021-02-24

**Authors:** Musa Ngayo

**Affiliations:** Kenya Medical Research Institute, Kenya

**Reviewer Prof. Lucy A. Semerjian**

 The research study investigates the microbial quality of selected bottled waters marketed in Kenya as well displays survey results from water bottling companies.

**Comment:** The title needs revising as it is as a conclusion in its current language.

**Response:** title changed to read. Contamination of bottled water brands with multidrug-resistant bacteria in Nairobi, Kenya.

**Comment:** The study is a basic investigation of microbial contamination in bottled water, drug resistance patterns of encountered microbes, and data collection on practices and processes of water bottling companies.

 Response: This is true

**Comment:** In the Methods section, the term "patients" is invalid and needs revising as the study is not related to patients.

**Respond:** The term patients dropped and replaced with study participants

**Comment:** The Sections” Results" and "Discussion" can be combined and restructured to avoid repetition of ideas and findings.

 Respond: The section maintained as separate but modified to minimize repeating results in the discussion section

**Comment:** Statistical analysis and conclusions could be more detailed and justified to give the research more scientific value.

**Response:** These sections have been clarified and improved where necessary

# Peer review: Reviewer response for version 1

**DOI:** 10.5256/f1000research.26509.r75673

**Published:** 2021-01-20

**Authors:** Ahmad Zarei

**Affiliations:** 1 Department of Environmental Health Engineering, School of Public Health, Social Development and Health Promotion Research Center, Gonabad University of Medical Sciences, Gonabad, Iran

This manuscript discusses Bottled water brands contaminated with multidrug resistant bacteria in Nairobi, Kenya. Water is essential for the human body and mental functions. This requires that water must be devoid of pathogens, dissolved toxins, and disagreeable turbidity, odor, color and taste. In my opinion, it is a good article to be accepted for indexing in F1000Research only after doing the following comments:

 Comments:

1. The English language of the paper should be improved.
2. For the paper title I propose use “Contamination of bottled water brands with multidrug resistant bacteria in Nairobi, Kenya” instead.
3. I think it is better to remove “role of water companies handling procedures” from “Keywords”.  
4. Reference 17 is before 1998. Please replace with a newer one (if possible).
5. Did the authors  consider the production date of the bottled water brands? If yes, how many bottles with different production dates were analyzed?
6. In Introduction. The authors can use the following paper:

- Chemical and microbial quality of bottled drinking water in Gonabad city, Iran: Effect of time and storage conditions on microbial quality of bottled waters

Is the work clearly and accurately presented and does it cite the current literature?

Yes

If applicable, is the statistical analysis and its interpretation appropriate?

Yes

Are all the source data underlying the results available to ensure full reproducibility?

Yes

Is the study design appropriate and is the work technically sound?

Yes

Are the conclusions drawn adequately supported by the results?

Yes

Are sufficient details of methods and analysis provided to allow replication by others?

Yes

Reviewer Expertise:

NA

I confirm that I have read this submission and believe that I have an appropriate level of expertise to confirm that it is of an acceptable scientific standard, however I have significant reservations, as outlined above.

## References

[1] : Chemical and microbial quality of bottled drinking water in Gonabad city, Iran: Effect of time and storage conditions on microbial quality of bottled waters. *MethodsX*.2019;6: 10.1016/j.mex.2019.02.001 273-277 10.1016/j.mex.2019.02.001 30805298PMC6374610

# Response

**Published:** 2021-02-24

**Authors:** Musa Ngayo

**Affiliations:** Kenya Medical Research Institute, Kenya

Response to reviewer’s comments

**Dr. Ahmad Zarei**

 Comments:

**Comment:** The English language of the paper should be improved.

 Respond: The paper has been re-read and the language moderated. We would that the reviewer points out exactly which section or how they would prefer the paper English language changed.

**Comment:** For the paper title I propose to use “Contamination of bottled water brands with multidrug-resistant bacteria in Nairobi, Kenya” instead.

 Respond: The title suggestion has been adopted

**Comment:** I think it is better to remove “role of water companies handling procedures” from “Keywords”.  

**Respond:** The key outcome of the paper actually addresses the role of water companies handling procedures on contamination of drinking water. We believe is key to this paper

**Comment:** Reference 17 is before 1998. Please replace it with a newer one (if possible).

**Response:** This reference by Lemeshow S, Hosmer DW Jr, Klar J: Sample size requirements for studies estimating odds ratios or relative risks. *Stat Med.* 1988;7(7):759–764. 3406603 10.1002/sim.4780070705 is still easily available online and describes sample size calculations for many scenarios including the formula adopted in this study. We are unable to access new reference

**Comment:** Did the authors consider the production date of the bottled water brands? If yes, how many bottles with different production dates were analyzed?

**Response**: All bottled water brands were of the same date and batch.

**Comment:** In Introduction. The authors can use the following paper:

**Response:** We have read the suggested paper but of the opinion that the introduction is adequate unless the reviewer has specific issues to be addressed
